# Systemic Activation of TLR3-Dependent TRIF Signaling Confers Host Defense against Gram-Negative Bacteria in the Intestine

**DOI:** 10.3389/fcimb.2015.00105

**Published:** 2016-01-12

**Authors:** Jose Ruiz, Saravana Kanagavelu, Claudia Flores, Laura Romero, Reldy Riveron, David Q. Shih, Masayuki Fukata

**Affiliations:** ^1^Division of Gastroenterology, Department of Medicine, University of Miami Miller School of MedicineMiami, FL, USA; ^2^Division of Gastroenterology, Department of Medicine, Cedars-Sinai Medical Center, F. Widjaja Foundation, Inflammatory Bowel and Immunology Research InstituteLos Angeles, CA, USA; ^3^Department of Medicine, David Geffen School of Medicine, University of CaliforniaLos Angeles, CA, USA; ^4^Department of Cell Biology, University of Miami Miller School of MedicineMiami, FL, USA; ^5^Department of Biomedical Science, Cedars-Sinai Medical CenterLos Angeles, CA, USA

**Keywords:** intestine, TIR-domain-containing adapter-inducing interferon-β (TRIF), defense mechanism, innate immunity, infection, natural killer cell

## Abstract

Recognition of Gram-negative bacteria by toll-like receptor (TLR)4 induces MyD88 and TRIF mediated responses. We have shown that TRIF-dependent responses play an important role in intestinal defense against Gram-negative enteropathogens. In the current study, we examined underlying mechanisms of how systemic TRIF activation enhances intestinal immune defense against Gram-negative bacteria. First we confirmed that the protective effect of poly I:C against enteric infection of mice with *Yersinia enterocolitica* was dependent on TLR3-mediated TRIF signaling by using TLR3-deficient mice. This protection was unique in TRIF-dependent TLR signaling because systemic stimulation of mice with agonists for TLR2 (Pam3CSK4) or TLR5 (flagellin) did not reduce mortality on *Y. enterocolitica* infection. Systemic administration of poly I:C mobilized CD11c+, F4/80+, and Gr−1^hi^ cells from lamina propria and activated NK cells in the mesenteric lymph nodes (MLN) within 24 h. This innate immune cell rearrangement was type I IFN dependent and mediated through upregulation of TLR4 followed by CCR7 expression in these innate immune cells found in the intestinal mucosa. Poly I:C induced IFN-γ expression by NK cells in the MLN, which was mediated through type I IFNs and IL-12p40 from antigen presenting cells and consequent activation of STAT1 and STAT4 in NK cells. This formation of innate immunity significantly contributed to the elimination of bacteria in the MLN. Our results demonstrated an innate immune network in the intestine that can be established by systemic stimulation of TRIF, which provides a strong host defense against Gram-negative pathogens. The mechanism underlying TRIF-mediated protective immunity may be useful to develop novel therapies for enteric bacterial infection.

## Introduction

In the intestine, host defense mechanisms drive effector immunity against pathogenic bacteria while regulating inflammatory responses to normal flora. This selection of host responses largely depends on pattern recognition receptors (PRRs), the germ-line encoded receptors that detect molecular components of microbial pathogens referred to as pathogen associated molecular patterns (PAMPs). Upon recognition of PAMPs, the cell is programmed to express a series of genes in order to mount an appropriate immune response to fight off the invading organism. Lipopolysaccharide (LPS), one of the PAMPs of Gram-negative bacteria, exists abundantly in the gastrointestinal lumen. Toll-like receptor 4 (TLR4) recognizes LPS and transduces intracellular signaling pathways through the adaptor molecules MyD88 or TRIF. Although TLR4 expression is down-regulated in normal intestinal mucosa, the TRIF pathway may have specific roles in intestinal homeostasis because its activation is restricted to TLR4 and TLR3, while the MyD88 pathway is induced by all TLRs except for TLR3. On the other hand, TLR3 exclusively induces the TRIF pathway in response to dsRNA viruses and is abundantly expressed in intestinal mucosa (Zarember and Godowski, 2002; Furrie et al., 2005; Monteleone et al., 2008). Therefore, exogenous activation of TLR3 may induce the TRIF-dependent host defense mechanism against Gram-negative pathogens in the intestine.

TRIF is a toll/interleukin-1 receptor-like (TIR) domain-containing adaptor protein that has been described to be a strong inducer of type I IFNs. Upon activation, TRIF induces activation of IRF3, NF-κB, and MAPK kinases resulting in unique gene expression. Recently, we have shown that TRIF-deficient (Trif^LPS2^) mice have impaired production of IFN-γ by natural killer (NK) cells in the MLNs and defective macrophage bactericidal function in response to enteric *Yersinia enterocolitica* infection (Sotolongo et al., 2011). We have also demonstrated that treatment of mice with synthetic dsRNA, poly I:C (TLR3 ligand), reduced mortality during enteric infection with *Y. enterocolitica*. These results suggest that TRIF could be an innovative therapeutic target to boost host defense against enteric pathogens. This effect of poly I:C also may be clinically relevant as most enteric pathogens are Gram-negative bacteria or viral pathogens that induce dsRNA during replication in the cytosol. However, it is important to determine the cellular and molecular mechanisms by which TRIF stimulation confers host defense in the intestinal mucosal interface. In addition, possible adverse effects of TRIF stimulation strategy should be taken into account. Poly I:C has been extensively studied as a potential immunologic adjuvant for viral infections, cancer immunotherapies, and effective immunizations, but has not been applied against bacterial infections (Verdijk et al., 1999; Longhi et al., 2009; Seya and Matsumoto, 2009; Tewari et al., 2010). Therefore, TRIF activation approach may have a broad applicability to variety of pathogens (Matsumoto et al., 2015).

The aim of this study is to dissect the underlying mechanism by which poly I:C-mediated TRIF activation protects host against enteric infection by Gram-negative pathogens. Systemic administration of poly I:C leads to a unique defense mechanism in the intestine that is mediated by mobilization of innate immune cells to the MLN from intestinal mucosa and induction of IFN-γ by NK cells in the MLN. This mobilization of innate immune cells requires TLR4 mediated CCR7 expression on these cells in the lamina propria and we show that systemic poly I:C increased expression of TLR4 in ileal mucosa. The effects of poly I:C are rapid and antigen non-specific and thus may be free from drug resistance. Therefore, fortifying innate immune defense by systemic activation of TRIF may be a reasonable strategy to fight against enteric infection with bacterial pathogens.

## Materials and methods

### Mice and procedures

WT C57BL/6J, Trif^LPS2^, and TLR4^−∕−^ mice were bred and housed under specific pathogen-free conditions in the Cedars-Sinai Medical Center animal facility. TLR3^−∕−^ mice were purchased from the Jackson Laboratory. All protocols were reviewed and approved by the Cedars-Sinai Institutional Animal Care and Use Committee. Poly I:C was subcutaneously (SC) injected (50 μg/mouse; TOCRIS Bioscience, Bristol, UK) and anti-IFNAR1 antibody (100 μg i.p/mouse, Leinco Technologies, St Lowis, MO) was administered at the same time. Flagellin (10 μg/mouse; Invivogen, San Diego, CA) and Pam3CSK4 (50 μg/mouse; Invivogen, San Diego, CA) were injected SC. Control mice received PBS. We used WT *Y. enterocolitica* (WA-314 serotype O:8) and *S. typhimurium* (SL1344) in this study. For infection studies, mice were orogastrically inoculated with *Y. enterocolitica* (1 × 10^7^ CFU/mouse) using a 22-gauge, round-tipped feeding needle (Fine Science tools; Echeverry et al., 2007).

### Cell preparation and purification

Single cell suspension of MLN was prepared by mechanical disruption with 70 μm nylon mesh. Peritoneal macrophages were isolated from peritoneal lavage as described previously (Sotolongo et al., 2011). WT NK (CD3-CD49+) and NKT (CD3+CD49+) cells from the MLN were purified by magnetic sorting with PE anti–mouse CD49 (DX5) and anti-PE Multi-Sort kit (Miltenyi Biotec, San Diego, CA). Lamina propria cells from small intestine were prepared as described previously (Kanagavelu et al., 2014). Briefly, the intestine was cut into small pieces and shaken at 200 r.p.m. for 20 min at 37°C with Ca^++^ Mg^++^ free Hank's balanced salt solution containing 5% fetal bovine serum (FBS) and 0.5 M EDTA. The pieces were further shaken at 200 r.p.m. for 60 min at 37°C with RPMI1640 containing 5% FBS, collagenase VIII (30 U/ml; Sigma, St Lowis, MO) and trypsin inhibitor (0.24 mg/ml; Sigma). Lamina propria cells were collected by filtering through a 70 μm cell strainer and then were purified with lymphocyte-separation medium (Cellgro, Corning, NY) by centrifugation at 800 g for 20 min at 20°C. LPS stimulation was carried out in a 96 well plate for 12 h (10 ng/ml) with a cell density of 2 × 10^5^/well. CD11c+, F4/80+, and Gr-1^hi^ cells were isolated by sorting using FACSAria III. Cells were stimulated with poly I:C (10 μg/ml) for 12 h.

### MLN bactericidal assay

MLN cells from mice injected with poly I:C or control mice were seeded with DME medium containing 10% FBS in a 96 well plate (2 × 10^5^ cells/well). MLN cells were then infected with *Y. enterocolitica* (MOI: 1) for 6 h at 37°C in the presence or absence of anti-IFNA1 antibody (10 μg/ml). MLN cells from control mice were treated with poly I:C (10 μg/ml) with or without anti-IFNA1 antibody (10 μg/ml) for 30 min then infected with *Y. enterocolitica* (MOI: 1) for 6 h at 37°C. MLN cells were lysed with 300 μl of distilled water and supernatants were plated on Yersinia specific agar plates. The same procedure was done for *S. typhimurium* (MOI: 10) infection in the presence of gentamicin (10 μg/ml). *S. typhimurium* associated with the MLN cells were collected by lysing with 300 μl of distilled water and samples were plated on LB agar plates. Data were expressed as fold changes over the results of the control samples.

### Cell staining and FACS analysis

Surface staining of CD11c, F4/80, Gr-1, CCR7, CD3, CD4, CD8, NK (CD3-CD49+), NKT (CD3+CD49+), B220, and pDCs (CD11c+B220+) intracellular staining of phosphor- STAT1 and STAT4, and IFN-γ were performed according to the manufacturer's instructions (eBioscience, San Diego, CA). GolgiPlug (BD) was added to the last 1.5 h of incubation. Alexa Fluor 488 conjugated anti-mouse γδTCR was purchased from BioLegend (San Diego, CA). FACS analyses were performed on an LSR II flow cytometer with FACS Diva (BD) and FlowJo (Tree Star).

### ELISA

NK cells were isolated from the MLN of WT mice as described earlier. Peritoneal macrophages isolated from WT mice were stimulated with poly I:C (10 μg/ml) for 24 h and supernatants were analyzed for IFN-β and IL-12p40 using ELISA duo kits according to the manufacturer's instructions (R&D Systems, Minneapolis, MN). For the IFN-γ production from MLN cells, MLN cells were isolated from mice 24 h post poly I:C injection and incubated for 24 h in a 96 well plate (2.5 × 10^5^/well). For the *in vitro* macrophage NK cell co-culture assay, peritoneal macrophages were pre-stimulated with poly I:C (10 μg/ml) for 6 h, and freshly isolated splenic WT NK cells were co-cultured for 24 h in the presence or absence of anti-IFNAR1 (10 μg/ml) or anti-IL-12 antibody (1 μg/ml, Peprotech). Supernatants were analyzed for IFN-γ using ELISA (R&D Systems).

### Real-time PCR

Total RNA was isolated from the small intestine using RNA Bee (Tel-Test, Friendwood, TX) according to the manufacturer's instructions. A total of 1 μg RNA was used as the template for single-strand complementary DNA synthesis using the QuantiTect Reverse Transcription Kit (Qiagen). Quantitative real-time PCR was performed for TLR4 (Sense: CAGCAGAGGAAGAACAAGAA and Antisense: TGCAAACAGACTGGGTTTAG), IFN-β (Sense: GTCCTCAACTGCTCTCCACT, and Antisense: GCAACCACCACTCATTCTG), IP-10 (Sense: TCCCTCTCGCAAGGAC, and Antisense: TTGGCTAAACGCTTTCAT), IFN-γ (Sense: GCTTGTACCTTTACTTCACTGAC, and Antisense: CTGGCCCGGAGTGTAGACAT), IL-18 (Sense: CCCAACGATAAAGAAGAACGCC and Antisense TGTCTGTGCCTCCCGTGCT GGC), TNF-α (Sense: GGAATGAGAAGAGGCTGAGACAT and Antisense CGTGGAACTGGCAGAAGAGG), and β-actin (Sense: TACGACCAGAGGCATACAG, and Antisense: ATGACCCAGATCATGTT TGA). The complementary DNA was amplified using Maxima SYBR Green/ROX (Thermo scientific, Pittsburgh, PA) on the Lightcycler (Roche, Indianapolis, IN). The amplification results of the genes of interest were normalized to the corresponding β-actin results and analyzed using the delta-delta Ct method.

### Statistical analysis

Kaplan-Meier survival curve was generated for infected mice, and statistical differences were analyzed by Chi square test. Student's *t*-test was used for two independent groups of samples. One-way ANOVA was used for more than two independent groups of samples, followed by Tukey's multiple comparison tests. All tests were performed with GraphPad Prism (Version 5.0b), and a *P*-value of < 0.05 was considered statistically significant.

## Results

### TLR3 is required for poly I:C mediated protection of mice from *Y. enterocolitica*

**Figure 1:**
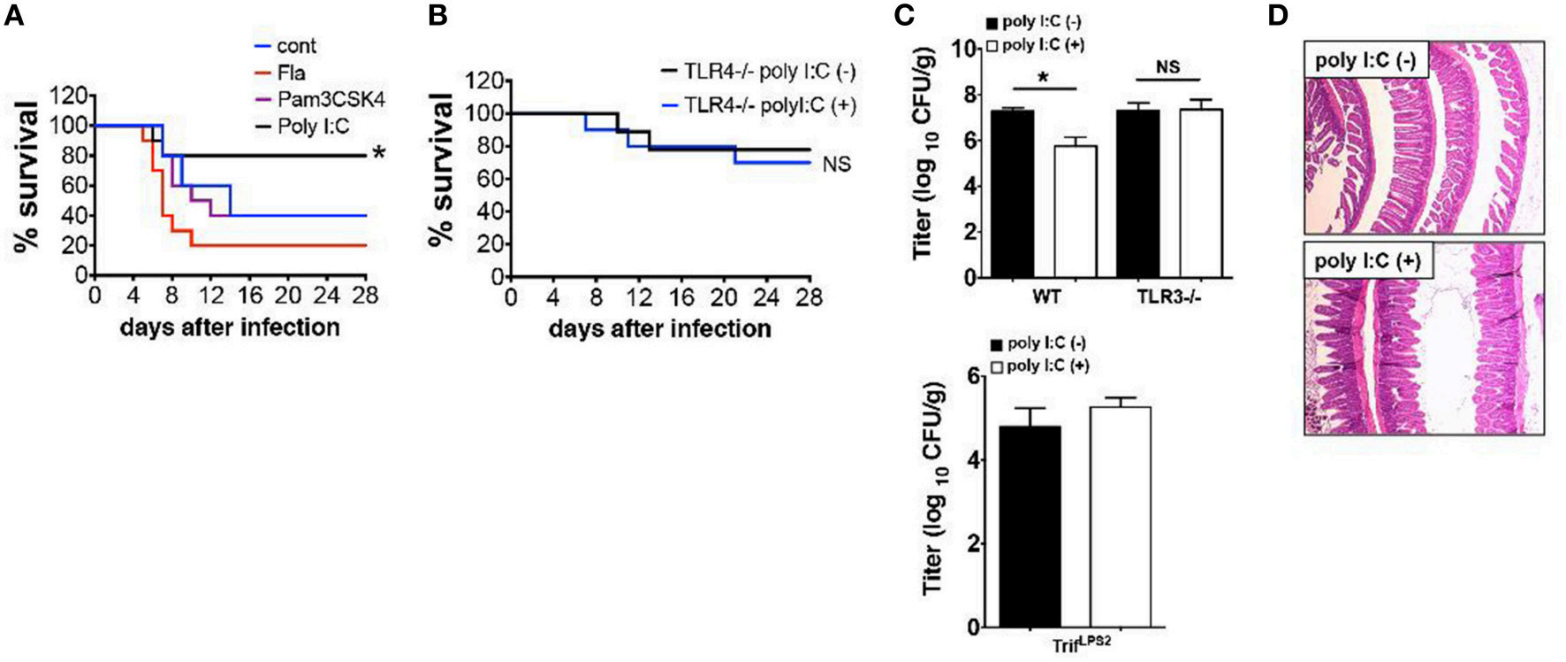
**Poly I:C protects mice from ***Y. enterocolitica*** infection, which depends on TLR3 and TLR4**. **(A)** Survival of WT mice during oral *Y. enterocolitica* infection (1 × 10^7^ CFU). Comparison of treatment among poly I:C, Fla and Pam3CSK4 (*n* = 10 each). **(B)** Survival of TLR4^−∕−^ mice during oral *Y. enterocolitica* infection (0.2 × 10^7^ CFU) with or without poly I:C (*n* = 5 each). **(C)** *Y. enterocolitica* titers in the MLNs of TLR3^−∕−^ mice SC injected with or without poly I:C followed by oral *Y. enterocolitica* infection (*n* = 6 each; day 7 post infection). Bottom graph shows *Y. enterocolitica* titers in the MLNs of Trif^LPS2^ mice 7 days post *Y. enterocolitica* infection (0.2 × 10^7^ CFU) with or without poly I:C (*n* = 6 each). **(D)** Histological analysis of the small intestine taken from WT mice 24 h after SC injection with poly I:C (800 μg/mouse). ^*^P < 0.05; NS, not significant. Error bars on graphs represent mean ± s.e.m.

We have shown that SC injection of poly I:C confers mice resistance to *Y. enterocolitica* infection through activation of TRIF signaling (Sotolongo et al., 2011). In this study, we sought to determine upstream mediators of TRIF signaling and whether this protection is TRIF-specific or can be reproduced by stimulation with other TLRs. Results demonstrated that systemic administration of Pam3CSK4 (TLR2 ligand) or Flagellin (ligand for TLR5 and NLRC4) did not result in improved survival as was seen with poly I:C injection (Figure 1A). Protective effect of poly I:C was not seen in TLR4^−∕−^ mice indicating an indirect contribution of TLR4 signaling to poly I:C mediated protection (Figure 1B). Of note, TLR4^−∕−^ mice received one-fifth *Y. enterocolitica* dose of WT experiments (Figure 1A) due to their higher susceptibility to the infection. Although poly I:C may stimulate retinoic acid-inducible gene 1 (RIG-I) and melanoma-differentiation associated gene 5 (MDA5) when introduced into cytosol, poly I:C mediated protection against *Y. enterocolitica* infection seems to be dependent on TLR3 mediated TRIF signaling as we could not observe the reduction of *Y. enterocolitica* growth in the MLN by poly I:C injection when we used TLR3^−∕−^ mice or Trif^LPS2^ mice (Figure 1C). We also addressed whether SC dose of poly I:C had negative influence in small intestinal mucosa because intraperitoneal injection of poly I:C has been shown to induce dosage dependent mucosal damage in the small intestine within 12 h (Zhou et al., 2007). No histologically detectable mucosal damage was found up to 800 μg/mouse poly I:C by SC injection (Figure 1D). Taken together, SC injection of poly I:C in a dose of 50 μg confers resistance to enteric *Y. enterocolitica* infection, which is mediated through TLR3 dependent TRIF signaling without disruption of intestinal mucosal integrity. Poly I:C mediated protection of mice from *Y. enterocolitica* infection also appear to involve TLR4 signaling.

### Systemic administration of poly I:C mobilizes innate immune cells from the lamina propria to the MLN

**Figure 2:**
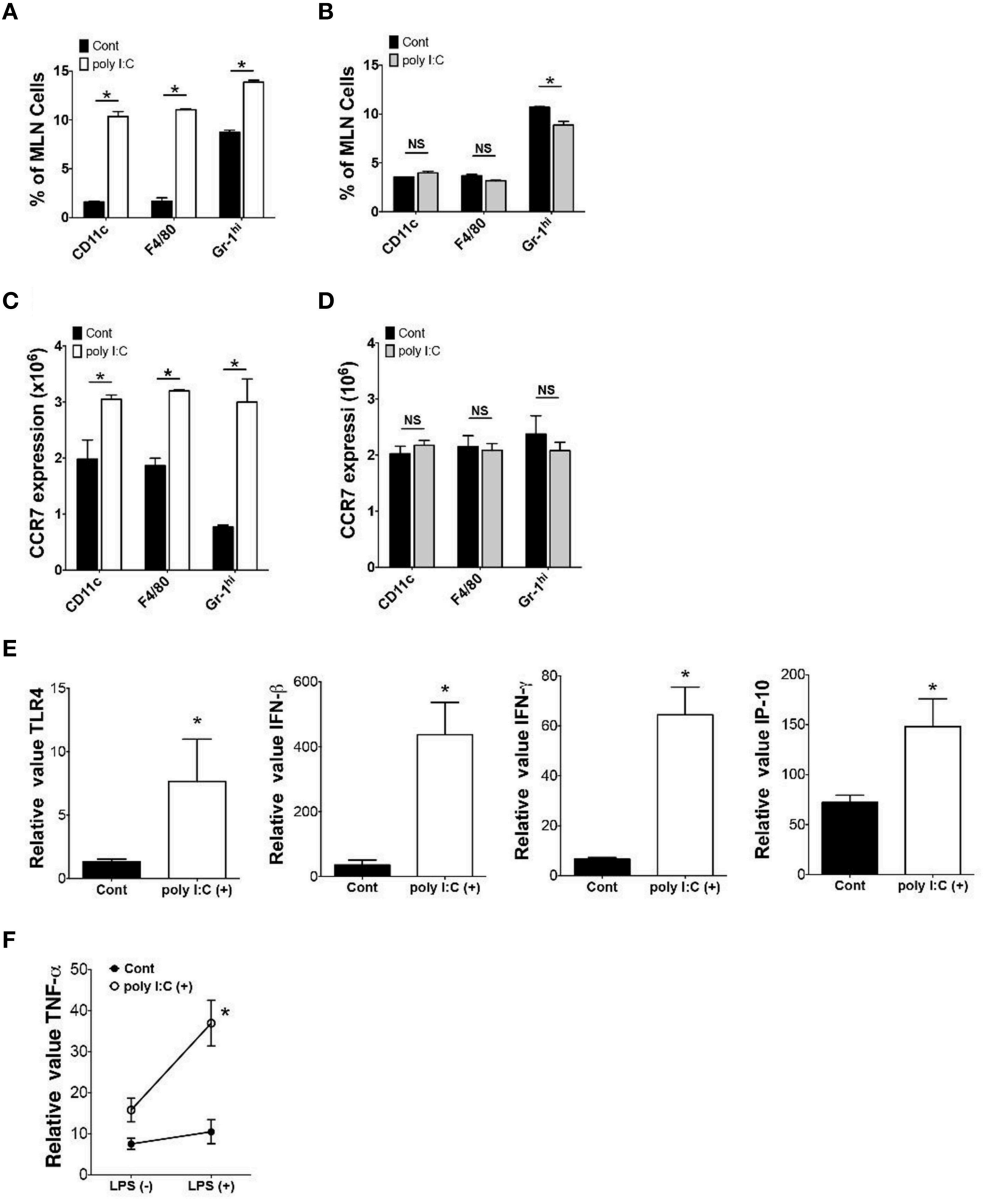
**Poly I:C mobilizes innate immune cells from lamina propria to MLN**. **(A,B)** FCM analysis of CD11c+, F4/80+ and Gr-1^hi^ cells in the MLN 24 h after poly I:C injection in WT or TLR4^−∕−^ mice (*n* = 3 each). **(C,D)** FCM analysis of CCR7 expression on CD11c+, F4/80+ and Gr-1^hi^ cells in the MLNs 24 h after poly I:C injection in WT or TLR4^−∕−^ mice (*n* = 3 each). **(E)** Real time PCR analysis of TLR4, IFN-β, IFN-γ, and IP-10 expression in the ileum of WT mice 24 h post poly I:C injection (*n* = 10 each). **(F)** Real time PCR analysis of LPS induced TNF-α expression in lamina propria cells isolated from WT mice with or without poly I:C injection (*n* = 4 mice each, LPS stimulation 10 ng/ml for 12 h). ^*^P < 0.05; NS, not significant. Error bars on graphs represent mean ± s.e.m.

To determine the underlying mechanisms of poly I:C-mediated protection of mice against enteric *Y. enterocolitica* infection, we examined alterations of cellular composition in the MLN 24 h after poly I:C injection. FCM analysis of cellular composition demonstrated an increased proportion of CD11c+, F4/80+, and Gr-1^hi^ cells in the MLN after poly I:C injection (Figure 2A). These alterations of cellular composition were not observed in TLR4^−∕−^ mice suggesting an involvement of TLR4 signaling in the mobilization of these innate immune cells (Figure 2B). Other cell types in the MLN did not show differences in proportion after SC poly I:C injection (Figure S1). Majority of mobilized DCs seemed to be myeloid DCs because increase of plasmacytoid DCs in the MLN after poly I:C injection was subtle (Figure 2A, Figure S1B). Because the MLN collects intestinal lymphatics, we addressed whether these innate immune cells were recruited from intestinal mucosa by detecting CCR7 expression in these cell types in the lamina propria (Figure 2C). We confirmed upregulation of CCR7 expression in CD11c+, F4/80+, and Gr-1^hi^ cells in small intestinal lamia propria 24 h after poly I:C injection (Figure 2C). Again, this upregulation of CCR7 in these innate immune cells in the lamina propria was not observed in TLR4^−∕−^ mice (Figure 2D). We confirmed that mRNA expression of TLR4 in ileal mucosa was upregulated after poly I:C injection (Figure 2E). In real time PCR analysis of ileum samples, the upregulation of IFN-β, IFN-γ, and IP-10 mRNA expression was also observed (Figure 2E). Poly I:C may induce IFN-γ through induction of IFN-β and IP-10 which in turn may induce upregulation of TLR4 expression (Nguyen et al., 2000; Tamai et al., 2003; Hold et al., 2011). This upregulation of TLR4 seemed to be functional as LPS induced TNF-α expression from lamina propria cells was significantly greater in poly I:C treated mice compared to control mice (Figure 2F). Because TLR4 signaling has been shown to induce CCR7 expression (Schmid et al., 2011), these results suggest that systemic activation of TLR3 mobilizes innate immune cells from lamina propria to the MLN through TLR4 mediated upregulation of CCR7, which may be associated with increased host resistance to enteric bacteria infection.

### Systemic poly I:C suppresses growth of gram-negative bacteria in the MLN

**Figure 3:**
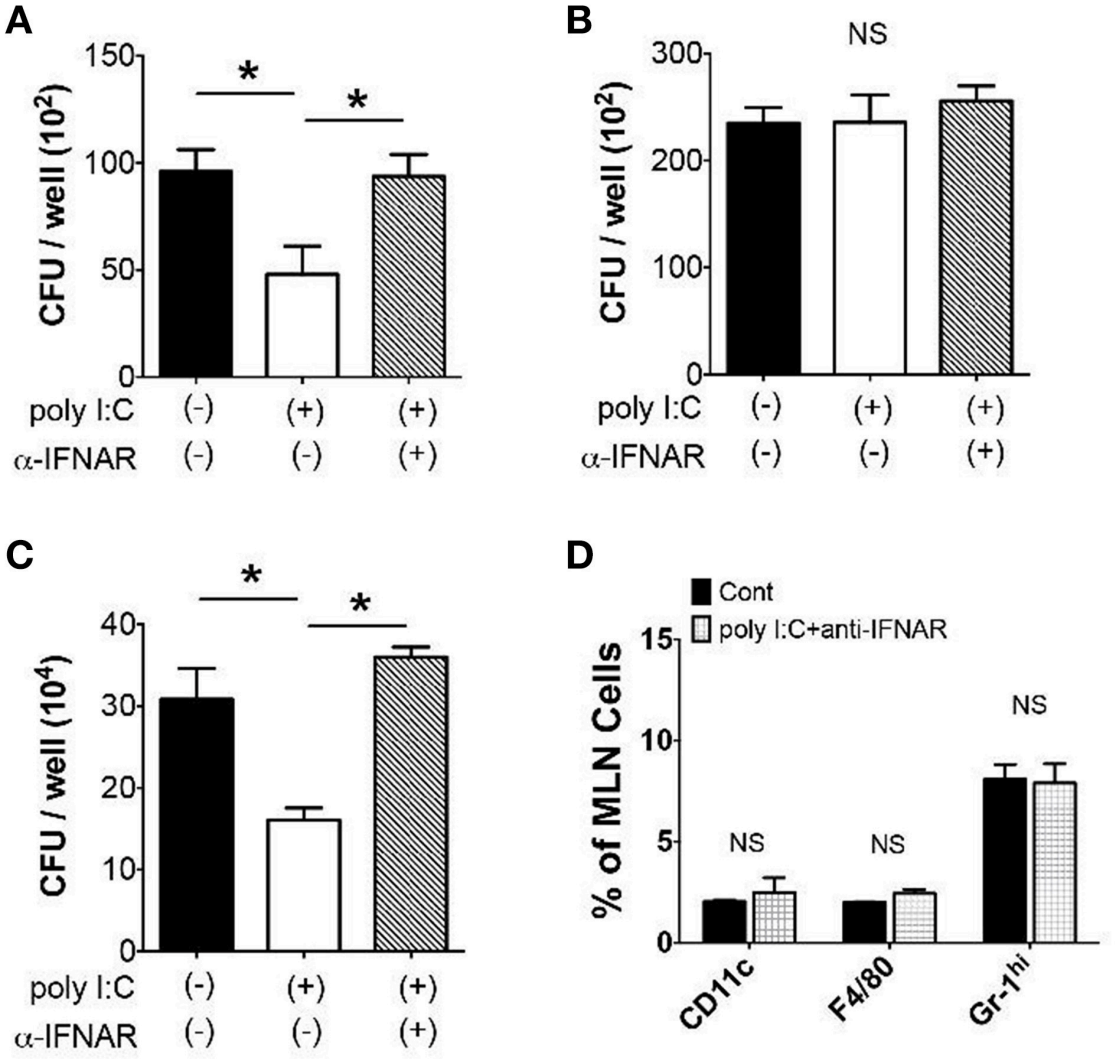
**Gram-negative bacterial growth in the MLN is suppressed by Poly I:C**. **(A,B)** *Y. enterocolitica* killing assay in MLN cells (2 × 10^5^/well, MOI: 1) taken from WT and TLR4^−∕−^ mice 24 h after poly I:C injection with or without α-IFNAR antibody (*n* = 10 each). **(C)** Intracellular *S. typhimurium* killing by the MLN cells (2 × 10^5^/well, MOI: 20) using gentamicin protection assay. The MLN cells were obtained from WT mice 24 h after poly I:C injection with or without α-IFNAR antibody (*n* = 10 each). **(D)** FCM analysis of CD11c+, F4/80+, and Gr-1^hi^ cells in the MLN taken from WT mice 24 h after poly I:C injection with or without α-IFNAR antibody (*n* = 4 each). ^*^P < 0.05; NS, not significant. Error bars on graphs represent mean ± s.e.m.

Given that poly I:C mobilizes innate immune cells to traffic into the MLN, we addressed whether these alterations in innate immune cell populations contributed host resistance to *Y. enterocolitica* infection. We found that MLN cells isolated 24 h post poly I:C injection suppressed *Y. enterocolitica* growth 50% more than MLN cells isolated from control mice (Figure 3A), and that this growth suppression was abolished by intraperitoneal administration of anti-IFNAR antibody together with SC poly I:C (Figure 3A). The reduction of *Y. enterocolitica* by MLN cells of poly I:C treated mice seems to be associated with TLR4-mediated mobilization of innate immune cells because we did not see this effect of poly I:C in TLR4^−∕−^ mice (Figure 3B). A similar effect of poly I:C was observed on intracellular bacterial killing of the MLN cells in a gentamicin protection assay using *S. typhimurium* (Figure 3C). In addition, the mice that received anti-IFNAR antibody did not show increased proportion of CD11c+, F4/80+, and Gr-1^hi^ cells in the MLN by poly I:C injection (Figure 3D). Therefore, systemic poly I:C leads to the mobilization of innate immune cells in the MLN in type I IFN and TLR4 dependent manner that facilitates elimination of bacteria in the MLN.

### Poly I:C suppresses bacterial growth in the MLN through NK cell activation

**Figure 4:**
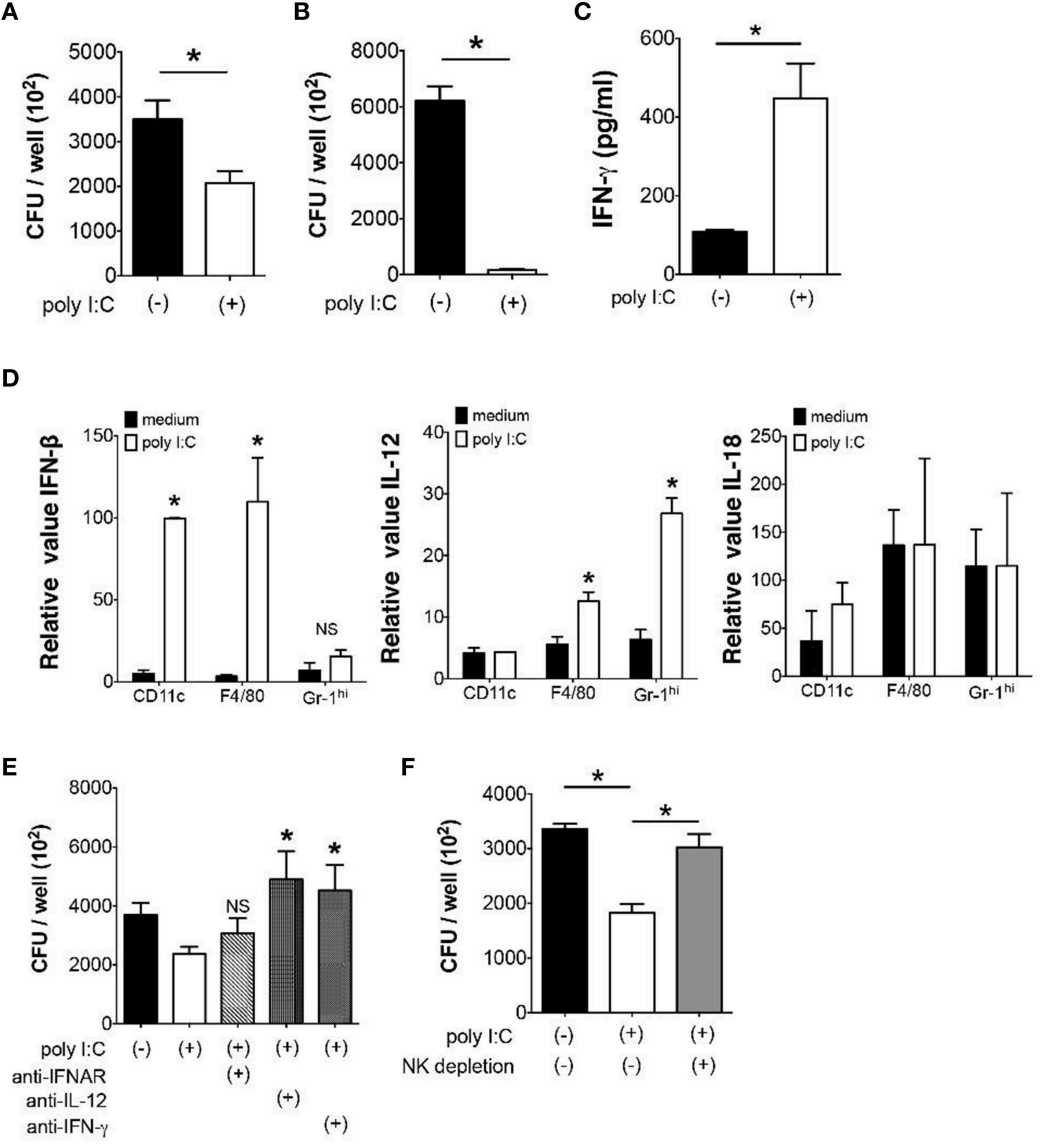
**Direct suppression of bacterial growth in the MLN by poly I:C requires NK cells**. **(A,B)** *In vitro Y. enterocolitica* killing assay using WT or TLR4^−∕−^ MLN cells in the presence or absence of poly I:C (*n* = 10 each). **(C)** ELISA analysis of IFN-γ production from MLN cells isolated from mice with or without poly I:C treatment (*n* = 4 mice each). **(D)** Real time PCR analysis of IFN-β, IL-12, and IL-18 expression from different innate immune cells of the MLN in response to poly I:C (*n* = 4 mice each, data from triplicated samples). **(E)** *In vitro* infection of MLN cells with *Y. enterocolitica* in the presence or absence of poly I:C together with neutralizing antibodies against IL-12, IFNAR, and IFN-γ (*n* = 6 each). **(F)** *In vitro Y. enterocolitica* killing assay in WT MLN cells that were depleted of NK cells. The effect of poly I:C in the presence or absence of α-IFNAR antibody (*n* = 10 each). ^*^P < 0.05; NS, not significant. Error bars on graphs represent mean ± s.e.m.

Next we examined whether poly I:C also has direct effect on bacterial elimination by MLN cells. When we examined *Y. enterocolitica* growth in MLN cells, a significant reduction of the number of *Y. enterocolitica* was observed in the presence of poly I:C (Figure 4A). Unlike *in vivo* effect of poly I:C, however, the direct effect of poly I:C on bacterial growth was independent of TLR4 signaling as we saw the full effect of poly I:C on MLN cells taken from TLR4^−∕−^ mice (Figure 4B).

Because IFN-γ is known to accelerate bacterial killing by phagocytes and poly I:C may induce IFN-γ production in variety of cells *in vivo*, we addressed whether poly I:C enhanced IFN-γ production by MLN cells (Schroder et al., 2004; Jiang et al., 2008). Supernatants of MLN cells that received poly I:C during *Y. enterocolitica* infection demonstrated significantly higher production of IFN-γ compared to supernatants of MLN cells without poly I:C (Figure 4C). In addition, the expression of IFN-β and IL-12 but not IL-18 mRNA was induced by poly I:C stimulation in CD11c+, F4/80+, and Gr-1^hi^ cells isolated from the MLN suggesting underlying mechanism of enhanced IFN-γ production by MLN cells (Figure 4D). Blocking IL-12 and IFN-γ during *Y. enterocolitica* infection in MLN cells actually showed significant reduction of bactericidal activity of MLN cells (Figure 4E). Blocking IFNAR had modest effect on bactericidal activity of MLN cells; differences were found neither between MLN cells nor MLN cells with poly I:C. The partial effect of IFNAR blockade may be due to the cell type difference in responses to type I IFNs.

Based on our finding that NK cell is important as a major source of IFN-γ for optimal elimination of Gram-negative bacteria in the MLN (Sotolongo et al., 2011), we tested bacterial growth in MLN cells after depletion of NK cells. Although we still found substantial growth suppression of *Y. enterocolitica* by poly I:C without NK cells, its effect was significantly lowered by depletion of NK cells (Figure 4F). These results indicate that poly I:C may directly facilitate bacterial killing in the MLN through induction of IL-12 and IFNs and activation of NK cells.

### Macrophages induce IFN-γ expression from NK cells through expression of type I IFNs and IL-12

**Figure 5:**
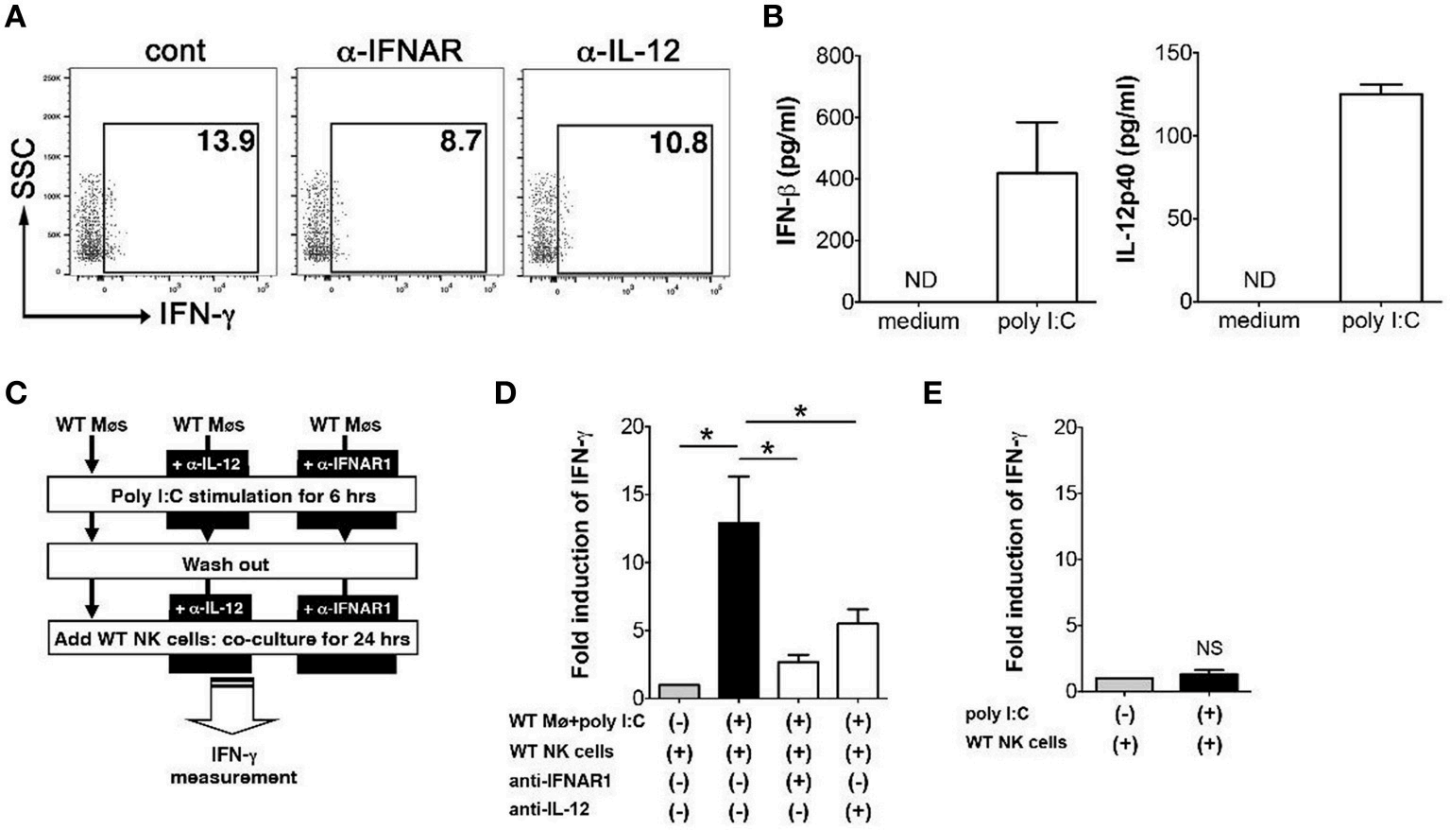
**Expression of type I IFNs and IL-12p40 by macrophages induces IFN-γ expression in NK cells**. **(A)** FCM analysis of IFN-γ expression from NK cells in the MLN; MLN cells were isolated from mice that received poly I:C and infected with *Y. enterocolitica* for 6 h (MOI: 1). **(B)** ELISA measurement of IFN-γ and IL-12p40 production from WT peritoneal macrophages stimulated with poly I:C for 24 h (*n* = 10 each). **(C,D)** Peritoneal macrophages from WT mice were stimulated with poly I:C for 6 h in the presence or absence of α-IFNAR or α-IL-12 antibodies. After washing out poly I:C, macrophages were co-cultured with WT splenic NK cells for 24 h and IFN-γ production was measured using ELISA (*n* = 10 each). **(E)** ELISA measurement of IFN-γ production by NK cells stimulated with poly I:C for 24 h (*n* = 10 each). ^*^P < 0.05; NS, not significant. Error bars on graphs represent mean ± s.e.m.

In order to determine how poly I:C activates NK cells, next we examined the mechanism of IFN-γ induction in NK cells that is mediated by poly I:C stimulated innate immune cells. We have shown an important role of IFN-β induced by macrophages in NK cell production of IFN-γ (Sotolongo et al., 2011). First we confirmed the contribution of type I IFNs and IL-12 to IFN-γ expression by NK cells in our system by FCM. Substantial reduction of IFN-γ expression from NK cells was observed during *in vitro Y. enterocolitica* infection of MLN cells when IFNAR or IL-12 were blocked (Figure 5A). In order to further address whether these cytokines expressed by poly I:C-stimulated macrophages are actually involved in IFN-γ expression from NK cells and if so what was the extent of the effect of each cytokine, we utilized mouse peritoneal macrophages based on their good response to poly I:C to induce protein production of IFN-β and IL-12 (Figure 5B). In addition to what we found earlier (Sotolongo et al., 2011), IFN-γ induction from NK cells was significantly suppressed by blockade of not only type I IFNs but also of IL-12 while the degree of suppression was greater in blockade of type I IFNs (Figures 5C,D). On the other hand, direct stimulation of NK cells with poly I:C did not induce IFN-γ expression (Figure 5E). These results indicate that poly I:C mediated activation of NK cells in the MLN is mediated by both type I IFNs and IL-12 that can be secreted from innate immune cells such as CD11c+, F4/80+, and Gr-1^hi^ cells.

### Poly I:C injection selectively enhances activation of STAT1 and STAT4 in NK cells in the MLN during *Y. enterocolitica* infection

**Figure 6:**
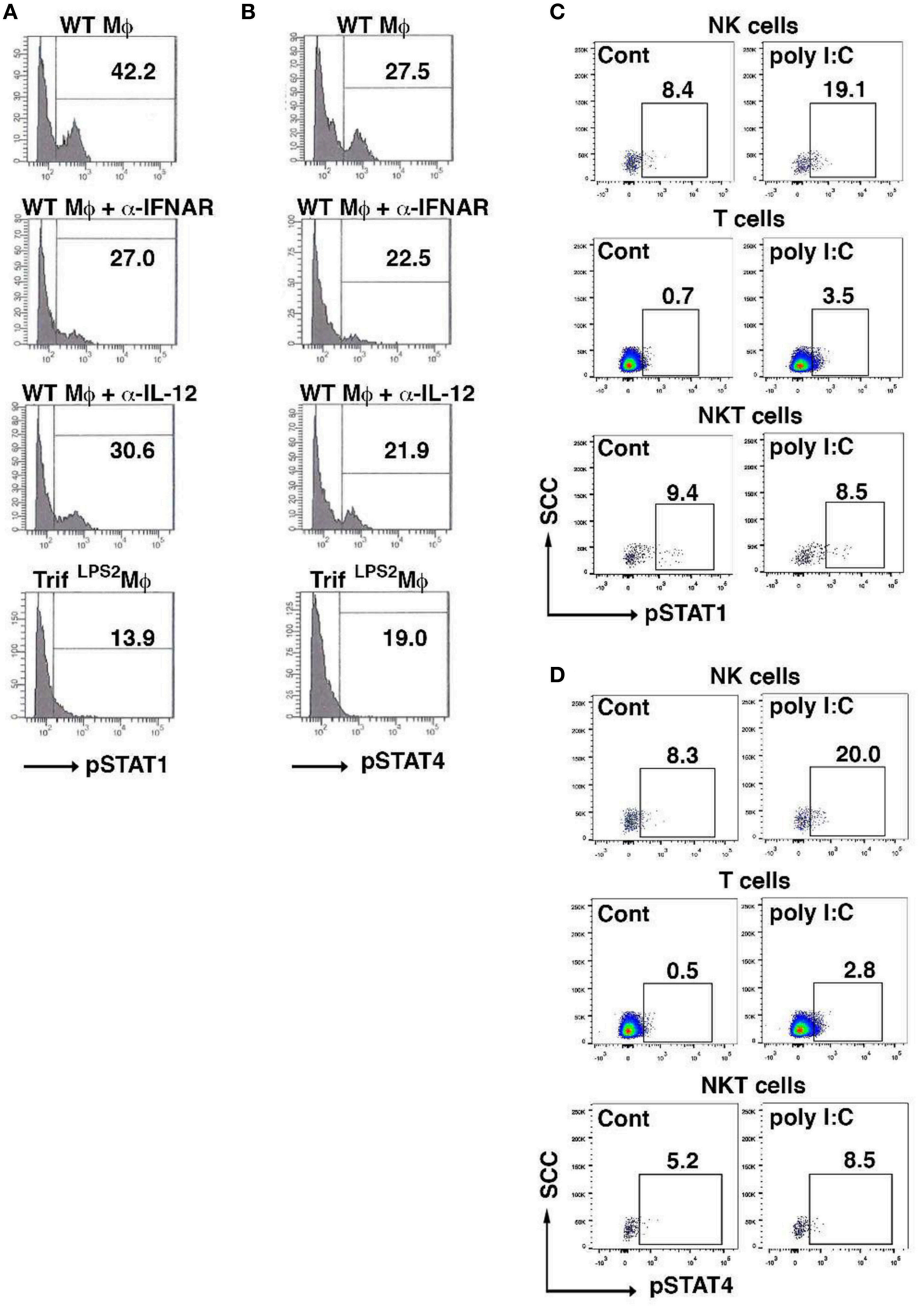
**Poly I:C activates STAT1 and STAT4 in NK cells in the MLN upon ***Y. enterocolitica*** infection**. **(A,B)** FCM analysis of phosphorylation of STAT1 and STAT4 in WT NK cells co-cultured with WT peritoneal macrophages 24 h after stimulation with poly I:C in the presence or absence of α-IFNAR or α-IL-12 antibody (*n* = 10 each). **(C,D)** FCM analysis of phosphorylation of STAT1 and STAT4 in NK, NKT, and T cells from the MLN obtained from WT mice 48 h after oral *Y. enterocolitica* infection (*n* = 4 each). The effect of poly I:C is shown. ^*^P < 0.05; NS, not significant. Error bars on graphs represent mean ± s.e.m.

Given both type I IFNs and IL-12 signaling are involved in NK cell induction of IFN-γ by poly I:C-stimulated macrophages, we examined how the IFN-γ induction in NK cells is transcriptionally regulated by analyzing phosphorylation of STAT1 and STAT4. Blocking type I IFNs as well as IL-12 during co-culture with poly I:C stimulated macrophages both reduced STAT1 phosphorylation in NK cells (Figure 6A). However, phosphorylation of STAT4 was relatively weak and only a modest suppression was seen by blocking type I IFNs or IL-12 (Figure 6B). As we have demonstrated that NK cells are major cell type expressing IFN-γ in the MLN during *Y. enterocolitica* infection, next we addressed whether poly I:C enhanced the phosphorylation of STAT1 and STAT4 in NK cells in the MLN during *Y. enterocolitica* infection. We found significantly increased phosphorylation of STAT1 and STAT4 in NK cells by poly I:C administration (Figures 6C,D). Slightly increased phosphorylation in both STAT1 and STAT4 was found in T cells but NKT cells demonstrated only minimally increased STAT4 phosphorylation by poly I:C administration (Figures 6C,D). These results suggest that NK cells are a dominant cell type that express IFN-γ in response to poly I:C in the MLN during enteric *Y. enterocolitica* infection, which requires activation of STAT1 and, to a lesser extent, STAT4.

## Discussion

The combined effects of globalization of the food supply and human travel have increased risks of enteropathogenic bacterial infections. Although antibiotic therapy may, in principle, be effective, the acquisition of antibiotic resistance to enteropathogens has become a serious clinical and public health concern (White et al., 2002; Walsh and Fanning, 2008). In the face of these issues, alternative strategies for preventing and treating such outbreaks are urgently needed. The severity of infections depends on proliferation of pathogens and the host immune responses, therefore, suppression of initial growth of pathogens by induction of a rapid immune response is crucial. In this regard, exploring innate immune signaling and regulation of protective immunity in the intestine is particularly significant. We have targeted TRIF signaling as a novel approach to tackle enteropathogenic infections based on our previous report that TRIF-deficient mice are susceptible to enteric infection with Gram-negative bacteria (Sotolongo et al., 2011). In the current study, we demonstrated the mechanisms by which systemic poly I:C administration facilitates elimination of infected Gram-negative enteropathogens in the MLN; poly I:C rearranged intestinal innate immune cells in the MLN by directly activating IFN signaling and indirectly through mobilization of innate immune cells from intestinal mucosa. Pathogens are less likely to induce resistance to this strategy because TRIF gene has been well conserved throughout the evolution and has not been overcome by any microorganisms.

Utilization of TLR signaling as a means to fortify host defense against pathogenic infections has been attempted. For example, exogenous stimulation of TLR5 and TLR9 has been assessed in mouse models of enteric *Salmonella* and vaginal Herpes simplex infections, respectively (Mariotti et al., 2002; Shen and Iwasaki, 2006; Vijay-Kumar et al., 2008). However, these efforts have been only partially successful because most TLR agonists produce strong adverse effects mainly induced by MyD88-dependent pro-inflammatory cytokines, and have not been approved for human use (Mata-Haro et al., 2007; O'Neill et al., 2009). In addition, the effect of TLR5 ligand on enteric bacterial infections might depend on the type of flagellin used as the previous report used Salmonella flagellin to fight against Salmonella infection (Vijay-Kumar et al., 2008) and we did not see the protective effect of flagellin on a different bacterial pathogen, *Y. enterocolitica*. By contrast, our results show that the TLR3 stimulation strategy is effective on multiple Gram-negative enteropathogens. TLR3 also has been shown to confer a defense against enteric protozoan infection (Lantier et al., 2014). TLR3-mediated TRIF signaling is unique in that it induces a distinct signaling pathway that mainly induces type I IFNs. The activation of this signaling by poly I:C has proven to be a potent adjuvant for immunotherapy against viruses, parasites, and cancers (Verdijk et al., 1999; Longhi et al., 2009; Seya and Matsumoto, 2009; Tewari et al., 2010). Therefore, in addition to boosting host innate defense mechanisms, the TRIF stimulation strategy may have universal applicability to enteric infection with many pathogens.

There are conflicting results in terms of the protective effect of poly I:C on the animal models of lung infections (Pyles et al., 2010; Tian et al., 2012). Intranasal administration of poly I:C enhanced bacterial clearance and extended survival in a mouse model of Gram-negative *F. tularensis* infection in the lungs (Pyles et al., 2010). On the other hand, pre-treatment with poly I:C resulted in impaired pulmonary clearance of secondary bacterial infections, particularly of Gram-positive *Methicillin-resistant Staphylococcus aureus* and *Streptococcus pneumoniae* (Tian et al., 2012). Therefore, the protective effect of poly I:C in infectious diseases may be restricted either to the gastrointestinal infections or Gram-negative bacterial infections in the case of other organs.

One of the mechanisms by which systemic administration of poly I:C induced protection against enteric bacterial infection was mobilization of innate immune cells in the MLN from intestinal mucosa. This mobilization was dependent on type I IFN signaling and TLR4-mediated CCR7 expression in these innate immune cells and was associated with mucosal expression of IFN-γ. Since TLR4 ligand LPS is abundantly available in intestinal lumen and poly I:C upregulated mucosal expression of TLR4, it is reasonable to think that systemic poly I:C increased sensitivity of TLR4 in intestinal mucosa. We showed increased TNF-α expression by lamina propria cells of the mice that received poly I:C in response to LPS. This is supported by the fact that poly I:C induced mucosal expression of IFN-γ as TLR4 sensitivity can be upregulated by IFN-γ (Abreu et al., 2002; Suzuki et al., 2003). The other mechanism involves expression of IFN-γ from activated NK cells in the MLN. We and others have demonstrated the requirement of antigen presenting cells in this activation of NK cells (Akazawa et al., 2007; Sotolongo et al., 2011). Our data further showed that both STAT1 and STAT4 activation in NK cells are involved in the induction of IFN-γ in NK cells. It is likely that multiple types of innate immune cells are involved in host defense mechanism against Gram-negative enteropathogens and their roles differ based on the location (Autenrieth et al., 2012; Sugiura et al., 2013). For example, lamina propria DCs might play an important role to establish adaptive immunity against locally invasive pathogens such as Salmonella enterica under the tight regulation with retinoic acid and TGF-β (Uematsu et al., 2008). Limitation of our study is that we used peritoneal macrophages to examine underlying mechanism of activation and IFN-γ secretion of NK cells in the MLN. Although we found significant increase of bactericidal activity in lamina propria cells isolated 24 h post poly I:C injection of mice (5.7 ± 0.1 poly I:C treated mice vs. 5.5 ± 0.1 non-treated mice, Log_10_CFU/2 × 10^5^ cells; MOI = 1 infected in 96 well plate for 6 h), the role of lamina propria cells in host defense against *Y. enterocolitica* infection has been obscure (Oellerich et al., 2007). Further research will help identify the effect of systemic poly I:C on lamina propria as well as splenic cells during enteric infection with Gram-negative bacterial pathogens.

In summary, our results demonstrated that subcutaneous administration of poly I:C induced protective immunity against enteric bacterial infection. This protective immunity was mediated at least in part by an innate immune interaction between intestinal mucosa and the MLN through sequential activations of TLR3 and TLR4 signaling that were linked by type I IFN signaling. This defense mechanism can be utilized to defend against multiple bacterial enteropathogens as it is mediated by antigen-nonspecific immunity and thus will not elicit drug resistance.

## Author contributions

JR performed *in vitro* infection FCM analysis and ELISA assays, and drafted manuscript, SK performed FCM analysis, real-time PCR, *in vivo* infection, and helped manuscript drafting, CF helped *in vitro* infection, FCM analysis, and real-time PCR analysis, LR maintained mouse colonies and performed RNA preparation, RR performed RNA preparation, helped *in vitro* infection and bacterial preparation, DS designed experiments and analyzed FCM data, MF designed experiments, performed *in vitro* infection and FCM analysis, executed all experiments, wrote manuscript.

### Conflict of interest statement

The authors declare that the research was conducted in the absence of any commercial or financial relationships that could be construed as a potential conflict of interest.

## Supplementary material

Figure S1 **Poly I:C does not mobilize other immune cells from lamina propria to the MLN. (A)** FCM analysis of CD3, CD4, CD8, NK, NKT, and B220 cells in the MLN 24 h after poly I:C injection in WT mice (*n* = 3 each). **(B)** FCM analysis of pDCs (CD11c+B220+), γδT cells in the MLN 24 h after poly I:C injection in WT mice (Left graph, *n* = 3 each) and TLR4^−∕−^ mice (Right graph, *n* = 3 each). NS, not significant. Error bars on graphs represent mean ± s.e.m.
