# Supplementary figures and images for: Systemic Activation of TLR3-Dependent TRIF Signaling Confers Host Defense against Gram-Negative Bacteria in the Intestine
Source: Front Cell Infect Microbiol. 2016 Jan 12;5:105. doi: 10.3389/fcimb.2015.00105 (PMC4710052; doi:10.3389/fcimb.2015.00105)

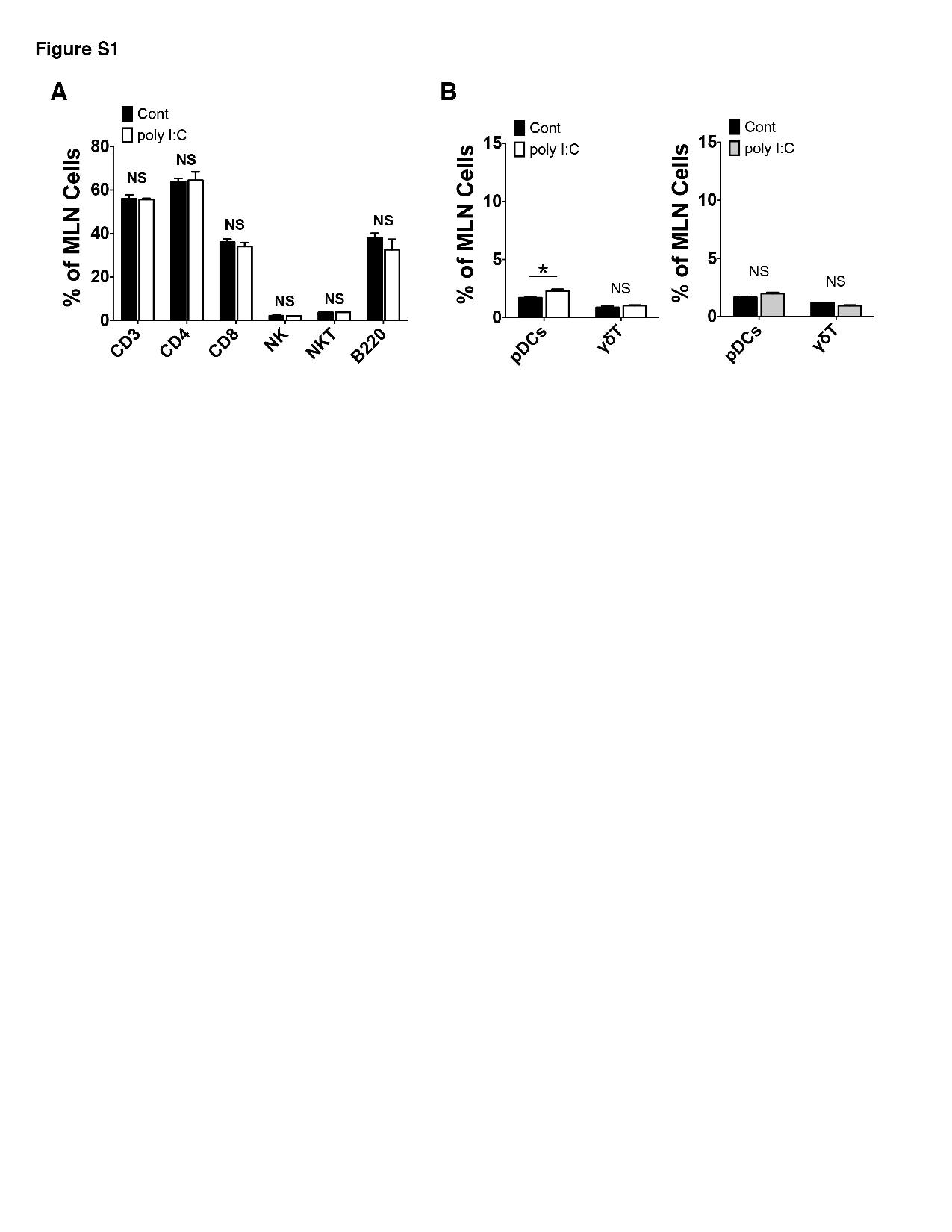

Supplement: Figure S1 — Poly I:C does not mobilize other immune cells from lamina propria to the MLN. (A) FCM analysis of CD3, CD4, CD8, NK, NKT, and B220 cells in the MLN 24 h after poly I:C injection in WT mice (n = 3 each). (B) FCM analysis of pDCs (CD11c+B220+), γδT cells in the MLN 24 h after poly I:C injection in WT mice (Left graph, n = 3 each) and TLR4−∕− mice (Right graph, n = 3 each). NS, not significant. Error bars on graphs represent mean ± s.e.m. [file Image1.JPEG]
